# Emergence of uncommon KL38-OCL6-ST220 carbapenem-resistant *Acinetobacter pittii* strain, co-producing chromosomal NDM-1 and OXA-820 carbapenemases

**DOI:** 10.3389/fcimb.2022.943735

**Published:** 2022-08-12

**Authors:** Chongmei Tian, Mengyu Xing, Liping Fu, Yaping Zhao, Xueyu Fan, Siwei Wang

**Affiliations:** ^1^ Department of Pharmacy, Shaoxing Hospital of Traditional Chinese Medicine Affiliated to Zhejiang Chinese Medical University, Shaoxing, China; ^2^ Department of Pharmacy, Affiliated Hangzhou First People’s Hospital, Zhejiang University School of Medicine, Hangzhou, China; ^3^ Department of Clinical Laboratory, Quzhou People’s Hospital, Quzhou Affiliated Hospital of Wenzhou Medical University, Quzhou, China; ^4^ Core Facility, Quzhou People’s Hospital, Quzhou Affiliated Hospital of Wenzhou Medical University, Quzhou, China

**Keywords:** Acinetobacter pittii, chromosomal NDM-1, OXA-820, Tn125, ST220, BSI

## Abstract

**Objective:**

To characterize one KL38-OCL6-ST220 carbapenem-resistant *Acinetobacter pittii* strain, co-producing chromosomal NDM-1 and OXA-820 carbapenemases.

**Methods:**

*A. pittii* TCM strain was isolated from a bloodstream infection (BSI). Antimicrobial susceptibility tests were conducted *via* disc diffusion and broth microdilution. Stability experiments of *bla*_NDM-1_ and *bla*_OXA-820_ carbapenemase genes were further performed. Whole-genome sequencing (WGS) was performed on the Illumina and Oxford Nanopore platforms. Multilocus sequence typing (MLST) was analyzed based on the Pasteur and Oxford schemes. Resistance genes, virulence factors, and insertion sequences (ISs) were identified with ABRicate based on ResFinder 4.0, virulence factor database (VFDB), and ISfinder. Capsular polysaccharide (KL), lipooligosaccharide outer core (OCL), and plasmid reconstruction were tested using *Kaptive* and PLACNETw. PHASTER was used to predict prophage regions. A comparative genomics analysis of all ST220 *A. pittii* strains from the public database was carried out. Point mutations, average nucleotide identity (ANI), DNA–DNA hybridization (DDH) distances, and pan-genome analysis were performed.

**Results:**

*A. pittii* TCM was ST220^Pas^ and ST1818^Oxf^ with KL38 and OCL6, respectively. It was resistant to imipenem, meropenem, and ciprofloxacin but still susceptible to amikacin, colistin, and tigecycline. WGS revealed that *A. pittii* TCM contained one circular chromosome and four plasmids. The Tn*125* composite transposon, including *bla*_NDM-1_, was located in the chromosome with 3-bp target site duplications (TSDs). Many virulence factors and the *bla*_OXA-820_ carbapenemase gene were also identified. The stability assays revealed that *bla*_NDM-1_ and *bla*_OXA-820_ were stabilized by passage in an antibiotic-free medium. Moreover, 12 prophage regions were identified in the chromosome. Phylogenetic analysis showed that there are 11 ST220 *A. pittii* strains, and one collected from Anhui, China was closely related. All ST220 *A. pittii* strains presented high ANI and DDH values; they ranged from 99.85% to 100% for ANI and from 97.4% to 99.9% for DDH. Pan-genome analysis revealed 3,200 core genes, 0 soft core genes, 1,571 shell genes, and 933 cloud genes among the 11 ST220 *A. pittii* strains.

**Conclusions:**

The coexistence of chromosomal NDM-1 and OXA-820 carbapenemases in *A. pittii* presents a huge challenge in healthcare settings. Increased surveillance of this species in hospital and community settings is urgently needed.

## Introduction

The genus *Acinetobacter* is ubiquitous in diverse environments and clinical settings. The species belonging to the *Acinetobacter calcoaceticus*–*Acinetobacter baumannii* complex (ACB complex) including *A. calcoaceticus*, *A. baumannii*, *A. dijkshoorniae*, *A. lactucae*, *A. nosocomialis*, *A. pittii*, and *A. seifertii* are of great importance (Sarshar et al., 2021). Among these species, *A. pittii* is an important opportunistic pathogen that mainly causes healthcare-associated infections, including bloodstream infections (BSIs), pneumonia, and urinary tract infections (UTIs) (Pailhories et al., 2018; Chopjitt et al., 2021). One important contributing factor to these nosocomial infections is their ability to survive in stressful environments and, consequently, how difficult they are to eradicate (Chapartegui-Gonzalez et al., 2022). These difficult to eradicate strains then lead to nosocomial infections, particularly in immunocompromised patients in intensive care units (Ding et al., 2022).

Carbapenems are still the main antimicrobial agents for the treatment of infections with multidrug-resistant *Acinetobacter* spp., including *A. pittii* (Hirschberg and Kopple, 1989; Bassetti et al., 2021). However, reports of carbapenem resistance are increasing recently and have created a huge therapeutic challenge for clinicians (Bonnin et al., 2014; Wang et al., 2016). Comprehensive understanding of these resistance mechanisms has been accelerated by advancements in whole-genome sequencing (WGS) technologies (Javkar et al., 2021).

Carbapenem resistance in *A. pittii* is mainly caused by class D carbapenemases such as OXA-23, OXA-40, and OXA-58 and, in some cases, by metallo-b-lactamases (MBLs), including the New Delhi metallo-β-lactamase (NDM) (Kaase et al., 2014). NDM could mediate resistance to most β-lactam antimicrobial agents, including penicillins, cephalosporins, and carbapenems (Feng et al., 2021). NDM in *A. pittii* strains was relevant to sporadic human infection with high mortality rates and hospital transmissions in various countries, and serves as a potential reservoir for the *bla*_NDM-1_-carrying plasmids. However, co-harboring of *bla*_NDM-1_ and *bla*_OXA-820_ in the chromosome of *A. pittii* has not been reported until now.

Apart from resistance, virulence also plays a key role in environmental persistence and the epidemic spread of disease (Chin et al., 2018). However, *A. baumannii* is commonly considered to be a low virulent pathogen (Chapartegui-Gonzalez et al., 2021), and studies regarding the virulence attributes of *A. pittii* are rarer (Cosgaya et al., 2019). Recently, a study from France described one case of *A. pittii* community-acquired pneumonia with a low virulence profile (Larcher et al., 2017). Considering the fact that various virulence factors can be found in *A. pittii* strains, studying its virulence and discussing its pathogenesis are still crucial in the pursuit of treating patients.

In this study, we investigate the characteristics of one sequence of type 220 A*. pittii* strain isolated from a bloodstream infection (BSI) in China. To the best of our knowledge, this is the first description of an ST220 *A. pittii* strain in which co-harboring *bla*_NDM-1_ and *bla*_OXA-820_ carbapenemase genes resided on the chromosome. A combination of Illumina and MinION whole-genome sequencing was conducted to provide comprehensive insight into the genomic and chromosome structure features.

## Materials and methods

### Flow chart indicating the process of this study

**Figure 1:**
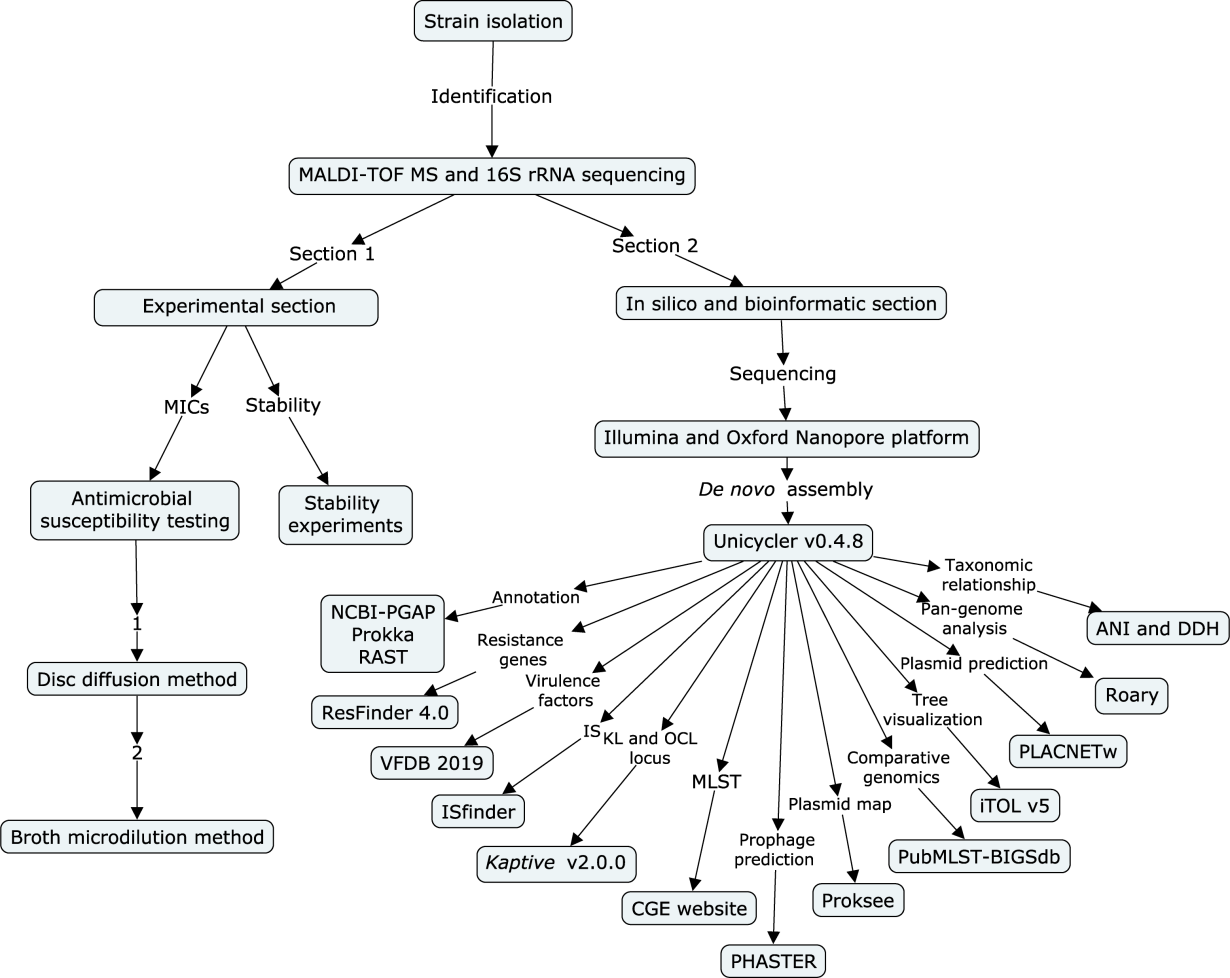
Flow chart of all experiments and employed procedures in this study. The flow chart was built using CmapTools v6.04. It is divided into an experimental section and a bioinformatic section.

A flow chart (**Figure 1**) was built to show all the experiments and employed procedures of this study using CmapTools v6.04 (https://cmap.ihmc.us) (Behzadi and Gajdacs, 2021). Based on the different parts of the experiments, sequencing, and bioinformatic sections, this study was divided into two parts (Ranjbar et al., 2017).

### Bacterial isolation, identification, and characterization of *A. pittii* isolate

*A. pittii* TCM strain was isolated from a BSI during routine diagnostic analysis on 19 January 2018 in Hangzhou, China. Isolate identification to species level was conducted by matrix-assisted laser desorption ionization–time of flight mass spectrometry (MALDI-TOF MS, Bruker Daltonik GmbH, Bremen, Germany) and confirmed by 16S rRNA gene-based sequencing (Nagy et al., 2009; Nagy et al., 2012).

### Antimicrobial susceptibility testing

Minimum inhibitory concentrations (MICs) for *A. pittii* TCM strain against multiple antibiotics were tested by using disk diffusion and broth microdilution. It interpreted breakpoints for *Acinetobacter* spp. according to the recommendations from the Clinical and Laboratory Standards Institute (CLSI), 2021 guidelines and European Committee on Antimicrobial Susceptibility Testing (EUCAST), 2021. Because there is no tigecycline breakpoint for *Acinetobacter*, MIC was interpreted according to the guidelines of EUCAST for *Enterobacterales* (with MIC breakpoint value ≤0.5 mg/L denoting susceptibility and >0.5 mg/L denoting resistance). The antibiotic disks used in this study included imipenem (IPM, 10 µg, Oxoid, Cheshire, UK), meropenem (MEM, 10 µg, Oxoid, Cheshire, UK), Amikacin (AMI, 30 µg, Oxoid, Cheshire, UK), and ciprofloxacin (CIP, 5 µg, Oxoid, Cheshire, UK) in a Mueller-Hinton agar (MHA) culture medium (Oxoid, Cheshire, UK). In addition to the antibiotics mentioned above, colistin (COL, Sigma-Aldrich, St. Louis, MO, USA) and tigecycline (TGC, Sigma-Aldrich, St. Louis, MO, USA) were also investigated using broth microdilution. *Escherichia coli* ATCC 25922 served as the quality control strain.

### Stability experiments of *bla*_NDM-1_ and *bla*_OXA-820_ carbapenemase genes

*A. pittii* TCM strain was grown overnight in three separate cultures at 37°C in 2 ml of Luria broth (LB) without antibiotics, followed by serial passage of 2 µl of overnight culture into 2 ml of LB daily, yielding 10 generations each lasting 7 days (Lv et al., 2020). On the last day, samples were collected and streaked on antibiotic-free MHA plates. Colonies were selected randomly, and the presence of *bla*_NDM-1_ and *bla*_OXA-820_ was confirmed *via* PCR. Primers were designed based on the full-length sequences of *bla*_NDM-1_ and *bla*_OXA-820_ on the *A. pittii* TCM chromosome (GenBank accession number: *CP095407*) using Snapgene (Dotmatics, USA) BLAST (NCBI, USA) 2.3.2 and BLAST software. Primers are listed in **Table S1**.

### Whole-genome sequencing and bioinformatics analysis

Genomic DNA was extracted from the *A. pittii* TCM strain using a Qiagen minikit (Qiagen, Hilden, Germany) in accordance with the manufacturer’s recommendations. Whole-genome sequencing was performed using both the Illumina HiSeq platform (Illumina, San Diego, CA, USA) and the Oxford Nanopore MinION platform (Nanopore, Oxford, UK). *De novo* assembly of the reads of Illumina and MinION was constructed using Unicycler v0.4.8 (Wick et al., 2017). The prediction and annotation of genome sequences were performed using the National Center for Biotechnology Information’s (NCBI) Prokaryotic Genome Annotation Pipeline (PGAP) updated in 2018 (Tatusova et al., 2016); Prokka v.1.13 (Seemann, 2014) and rapid annotations were done using the subsystems technology (RAST) server (Overbeek et al., 2014). Antimicrobial resistance genes were identified using the ABRicate v0.8.13 program (https://github.com/tseemann/abricate) based on ResFinder 4.0 updated in 2020 (http://genomicepidemiology.org/) (Zankari et al., 2012; Bortolaia et al., 2020). The point mutations were identified using the fIDBAC server and manual comparison (Liang et al., 2021). Bacterial virulence factors were identified through the virulence factor database updated in 2019 (VFDB 2019, http://www.mgc.ac.cn/VFs/) (Liu et al., 2022). Insertion sequences (ISs) were identified using the Isfinder updated in 2022 (Siguier et al., 2006). Capsular polysaccharide (K locus, KL) and lipooligosaccharide (OC locus, OCL) were tested using *Kaptive* v2.0.0 updated in 2021 (Wyres et al., 2020; Lam et al., 2022). Multilocus sequence typing (MLST) was performed *via* the Center for Genomic Epidemiology (CGE) website updated in 2020 (https://cge.cbs.dtu.dk/services/MLST/). The Phage Search Tool (PHASTER) updated in 2016 was used for the prediction of bacteriophages (Zhou et al., 2011; Arndt et al., 2016).

Plasmid reconstruction was conducted using the PLACNETw tool (Vielva et al., 2017). Plasmid structure was visualized using Proksee (https://proksee.ca/) (Stothard et al., 2019). Comparative genomics analysis of all 11 ST220 *A. pittii* strains from the PubMLST database (https://pubmlst.org/) was further performed using the Bacterial Isolate Genome Sequence Database (BIGSdb) (Jolley et al., 2018). The generation tree file was visualized using the Interactive Tree of Life updated in 2021 (iTOL v5, https://itol.embl.de/) (Letunic and Bork, 2021). Default parameters were used for all software packages.

The taxonomic relationships among these isolates were further evaluated using the average nucleotide identity (ANI) (Luis and Konstantinos, 2016) and DNA–DNA hybridization (DDH) distances (Meier-Kolthoff et al., 2013), with *A. pittii* HUMV-6483 as the reference strain and species positive control (Chapartegui-Gonzalez et al., 2017). Pan-genome analysis was performed *via* the Roary software (Page et al., 2015) and visualized using Phandango (https://jameshadfield.github.io/phandango/#/main).

## Results

### Genome annotations and subsystem categories

The genome and protein-coding sequences (CDS) were annotated and predicted using PGAP and RAST. According to PGAP annotation, there are 4,330 genes, of which 4,102 are protein-coding genes, 131 are pseudogenes, and the remaining 97 are predicted RNA-coding genes, composed of 74 tRNAs, 18 rRNAs and 5 ncRNAs.

**Figure 2:**
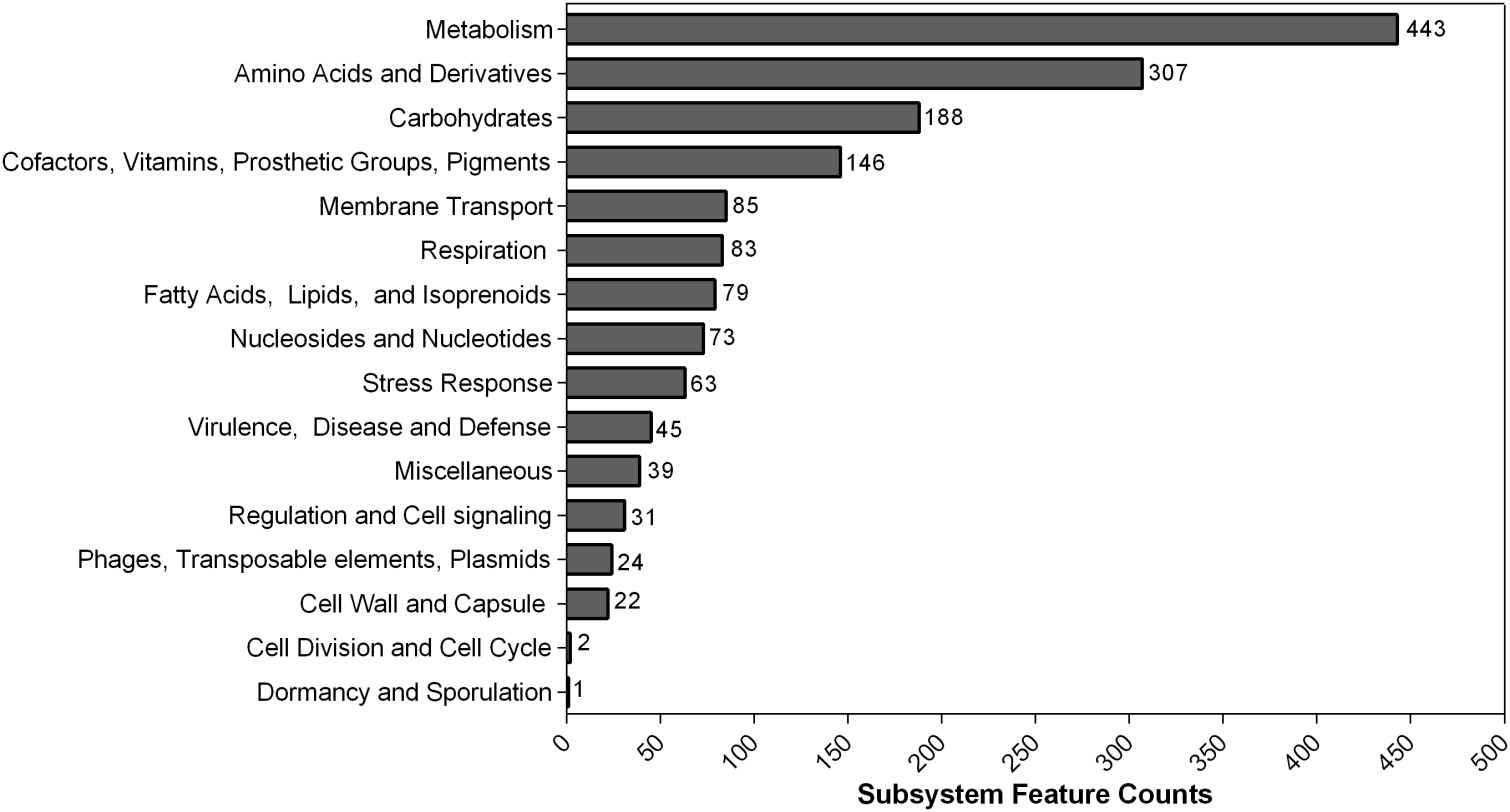
Counts of protein-coding sequences distributed in each subsystem feature based on RAST annotation. The number of each subsystem category is shown on the right of each column.

In contrast to PGAP, 4,280 genes that belonged to 313 subsystems were annotated using RAST. The subsystem each CDS was classified into is shown in **Figure 2**. Most of them belonged to metabolism (443) and amino acids and derivatives (307). Additionally, 45 CDS were sorted into virulence, disease, and defense categories.

### Susceptibility to antimicrobial agents

The antimicrobial susceptibility testing results revealed the *A. pittii* TCM strain possessed a multidrug-resistant (MDR) profile when both CLSI and EUCAST breakpoints were used. The inhibition zone diameters of imipenem, meropenem, ciprofloxacin, and amikacin were 17 mm (R), 13 mm (R), 7 mm (R), and 21 mm (S), respectively. The broth microdilution results showed that the MICs of imipenem and meropenem were 8 and 16 mg/L, respectively. *A. pittii* TCM strain also exhibited a resistance to ciprofloxacin (32 mg/L). In our case, *A. pittii* TCM was still susceptible to amikacin (2 mg/L), colistin (1 mg/L), and tigecycline (0.5 mg/L). The susceptibility of the *A. pittii* TCM strain against the antimicrobial agents above was consistent when the isolate was classified as resistant or susceptible using CLSI and EUCAST breakpoints.

### Antimicrobial resistance determinants, point mutations, and virulence profiles

Analysis of the genome of the *A. pittii* TCM strain revealed that in addition to co-harboring *bla*_NDM-1_ and *bla*_OXA-820_, a series of genes conferring resistance to β-lactams (blaADC-43), bleomycin (*ble-MBL*), streptomycin [*ant(2’’)-Ia*], sulfonamides (*sul2*), and macrolide [*msr(IE)* and *mph(E)*] were also identified (**Table 1**). Regarding fluoroquinolones resistance, serine by lysine at position 81 (S81L) in the *gyrA* (DNA gyrase) was found. However, no *pmrAB* and *lpxACD* mutations were identified in this strain. Based on these results, the genotype and the phenotype were consistent.

**Table 1:** Molecular characterization of genomes from the *A. pittii* TCM strain.

Genome	Size(bp)	GCcontent	Resistancegenes	Accession numbers
chromosome	4,250,902	39.00%	*bla*_NDM-1_, *bla*_OXA-820_, blaADC-43, *ble-MBL*,	*CP095407*
pTCM-1	84,108	39.54%	sul2	*CP095408*
pTCM-2	11,346	33.29%	ND	*CP095409*
pTCM-3	8,505	35.49%	msr*(E)*, mph*(E)*	*CP095410*
pTCM-4	6,078	39.19%	*ant(2’’)-Ia*	*CP095411*

ND, not detected.

Many virulence factors were identified in the *A. pittii* TCM strain. One was the outer membrane protein *ompA* gene *pga* operon (*pgaABCD*) encoding poly-β-1,6-N-acetyl-d-glucosamine (PNAG), which is important for biofilm development. Others were *csu* operon encoding Csu pili and *pbpG* encoding PbpG for serum resistance—a quite important two-component regulatory system *bfmRS* involved in Csu expression. Then, finally, there were *lpxABC* and *lpxL* encoding lipopolysaccharide (LPS) and many genes (*bauABCDEF*, *basABCDEFGHIJ*, and *barAB*) encoding Acinetobactin for iron uptake.

### Stability of *bla*_NDM-1_ and *bla*_OXA-820_

The stability assays revealed that *bla*_NDM-1_ and *bla*_OXA-820_ were quite stable even after 70 samples under antibiotics-free condition. These results were confirmed with PCR (**Supplementary Figure 1**).

### Multilocus sequence typing, lipooligosaccharide outer core, and capsular polysaccharide

**Figure 3:**
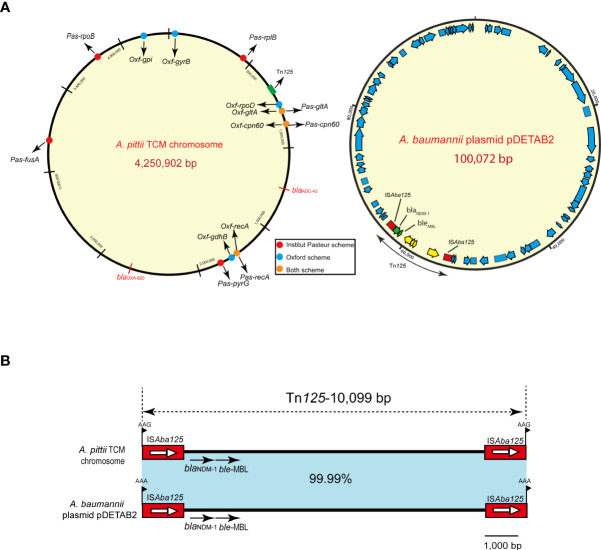
*A. pittii* TCM strain chromosome circular map and Tn*125* composite transposon structure. **(A)** Circular map of the *A. pittii* TCM chromosome. Housekeeping genes of Pasteur and Oxford schemes are indicated by various colors. On the right panel of **(A)**, *A. baumannii* plasmid pDETAB2 and Tn*125* composite transposon structure are shown. **(B)** Structure of Tn*125* composite transposon compared with plasmid pDETAB2 (CP047975). Horizontal arrows represent the direction of the genes, with black arrows indicating resistance genes. Light blue shades indicate regions with 99.99%–100% identity. Target site duplication (TSD) is shown as flag in black.

Based on the Pasteur MLST scheme, the *A. pittii* TCM strain was typed into a sequence type 220 (*cpn60*-45, *fusA*-20, *gltA*-44, *pyrG*-16, *recA*-20, *rplB*-29, *rpoB*-20). According to the Oxford MLST schemes, it belonged to ST1818 (*cpn60*-52, *gdhB*-141, *gltA*-62, *gpi*-247, *gyrB*-129, *recA*-7, *rpoD*-10). The specific positions of all housekeeping genes are shown in **Figure 3A**.

*Kaptive* showed that the *A. pittii* TCM strain contains OC locus 6 (OCL-6), matching the 98.98% coverage of reference sequence with 80.55% nucleotide identity. The K locus in the *A. pittii* TCM strain is KL38. It matches 100% of the locus with an overall nucleotide identity of 96.89%.

### Chromosome and Tn*125* composite transposon structure

The hybrid assembly of Illumina and MinION reads showed that the *A. pittii* TCM strain had a 4,250,902-bp circular chromosome (**Figure 3A**) with guanine cytosine (GC) content of 39.00% (**Table 1**).

The *bla*_NDM-1_ gene cluster was arranged sequentially as IS*Aba125*, *bla*_NDM-1_, and *ble-MBL* elements (**Figure 3B**). The *bla*_NDM-1_ gene was embedded in the composite transposon Tn*125*, bracketed by two copies of the IS*Aba125* orientated in the same direction, and flanked by 3-bp (AAG) possible target site duplications (TSDs). Moreover, the composite transposon structure of Tn*125* with another 3-bp (AAA) TSD in the plasmid pDETAB2 (GenBank accession number: CP047975), which was isolated from a rectal swab sample in China, is identical to this Tn*125* with a percentage of 99.99% (**Figure 3B**).

### Plasmid prediction and genetic analysis of plasmids

**Figure 4:**
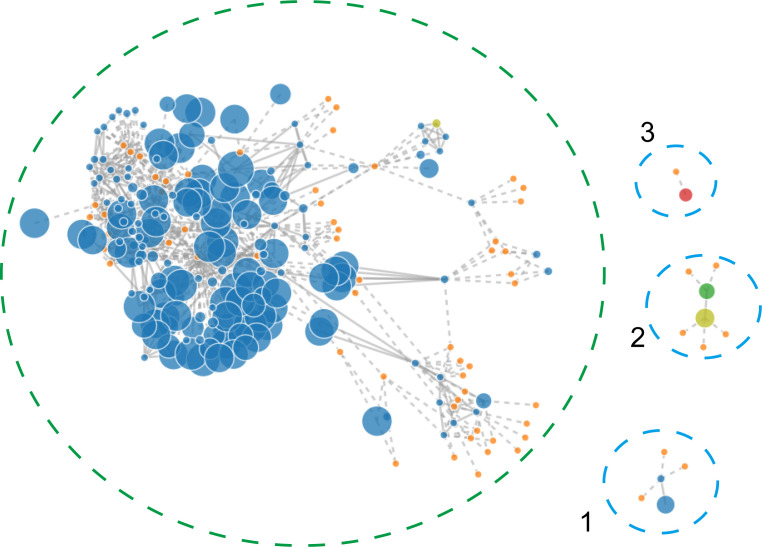
PLACNETw was used for plasmid reconstruction from the A. pittii TCM strain draft genomes. The contigs node’s sizes are proportional to their length. Blue nodes represent contigs of *A. pittii* TCM genomes, and orange nodes indicate the reference genomes. Contigs nodes’ sizes are proportional to their length. Color nodes represent contigs with special function proteins: yellow-green (replication initiation protein), red (relaxases), and green represents both. The green-dotted line shows the chromosome and the three blue-dotted lines show the plasmids.

To identify the contigs that may belong to a plasmid, the PLACNETw software was used based on the Illumina sequencing data. Result showed that the *A. pittii* TCM strain had a chromosome of 3.9 Mb and three predicted plasmids with 20.22, 11.45, and 6.27 kb, respectively (**Figure 4**).

**Figure 5:**
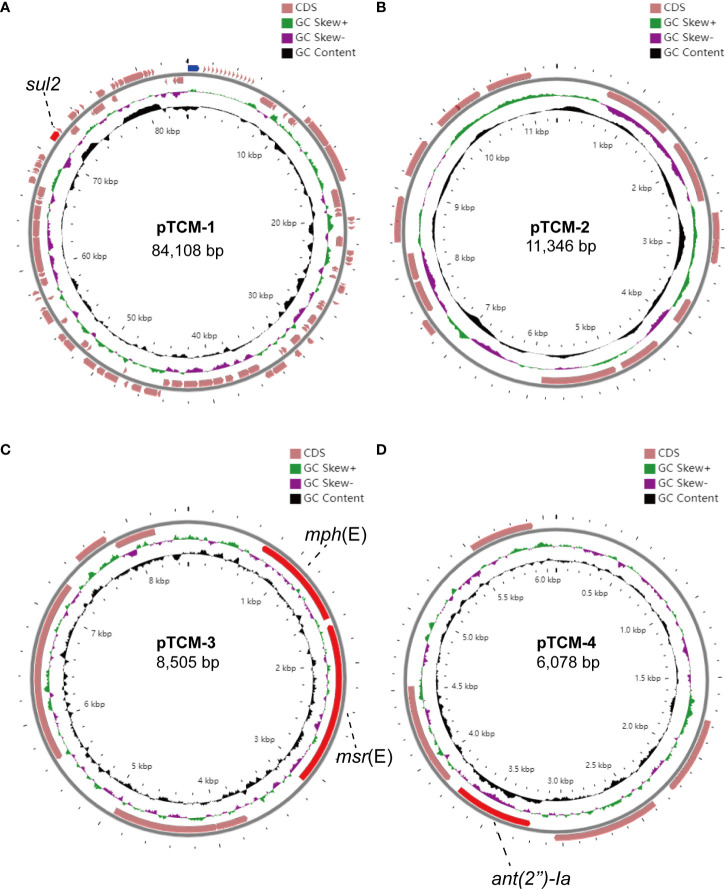
Four circular plasmid maps in *A. pittii* TCM strain. Red arrows indicate the antimicrobial resistance genes. Pink arrows indicate the other open reading frames (ORFs). Antimicrobial resistance genes carried by the plasmid are labeled. **(A)** Circular plasmid map of pTCM-1. **(B)** Circular plasmid map of pTCM-2. **(C)** Circular plasmid map of pTCM-3. **(D)** Circular plasmid map of pTCM-4.

We further assembled the complete genome with both short-read Illumina sequencing data and long-read Oxford Nanopore sequencing data. To visualize the plasmid maps of the *A. pittii* TCM strain, a member of the CGView (Circular Genome Viewer) software family, Proksee, was utilized for generating high-quality, navigable maps of circular genomes. This tool is a Java program and originally intended for bacterial genomes. In our case, four plasmids were identified in the *A. pittii* TCM strain, namely pTCM-1 to pTCM-4, with sizes between 6,078 and 84,108 bp and GC contents ranging from 33.29% to 39.54% (**Table 1**). Different kinds of resistance genes were carried by pTCM-1, pTCM-3, and pTCM-1 and showed in the plasmid maps (**Figures 5A, C, D**). However, pTCM-2 does not carry any resistance genes (**Figure 5B**).

### Prophage regions in the chromosome

**Figure 6:**
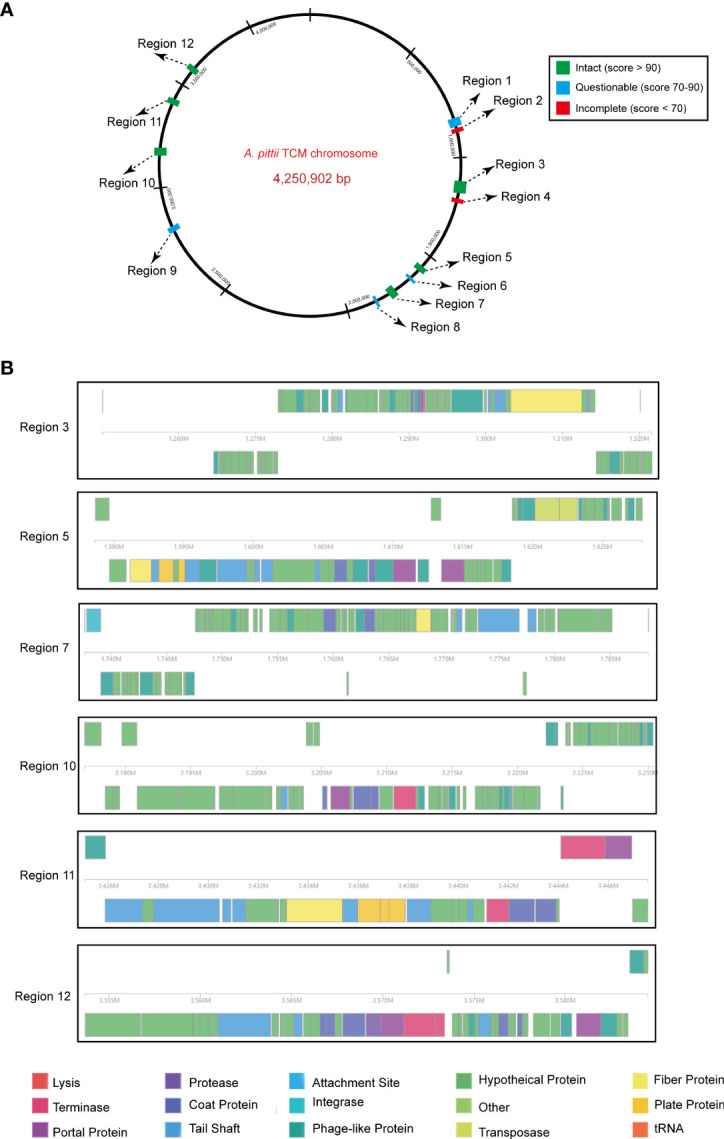
Predicted prophage regions within the *A*. *pittii* TCM chromosome. **(A)** A total of 12 prophage regions are positioned in the chromosome. Green means the intact prophage regions (score >90), blue means the questionable prophage regions (score 70–90), and red means the incomplete prophage regions (score <70). **(B)** Structure of six intact prophage regions. Genes are colored based on predicted functions.

Prophage regions were predicted by the PHASTER tool. The results showed six intact, four questionable, and two incomplete regions in the chromosome (**Figure 6A**). Regions 3, 5, 7, 10, 11, and 12 were 71.5, 39.1, 50.8, 43.4, 22.5, and 30.8 kb long with GC contents of 39.27%, 40.92%, 38.10%, 39.60%, 38.25%, and 40.27%, respectively. Based on the PHASTER tool, all six regions above were predicted to be intact due to having scores of >90. The gene function of the six intact prophage regions, known to be essential for phage activity such as specifying attachment, structural components, phage integration, and cell lysis were identified (**Figure 6B**). In addition, regions 1, 6, 8, and 9 were classified as questionable due to their scores of 70 or 80. However, there were also two incomplete prophage regions with scores of 20 and 30, respectively.

### Comparative genomics analysis of all ST220 *A. pittii* strains in public database

**Figure 7:**
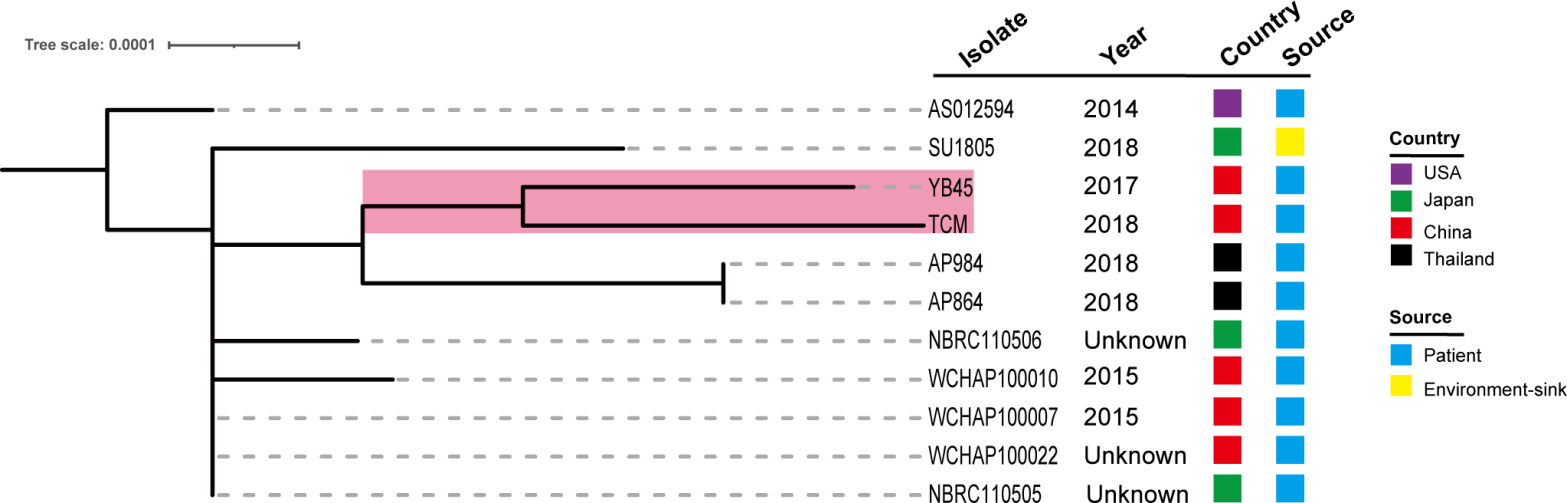
Phylogenetic analysis of 11 ST220 *A. pittii* strains based on the public database. The tree was built *via* the PubMLST database and visualized with iTOL v5. Isolates name, isolation date, country, and collection sources are shown in each isolate. The filled boxes reveal the different countries or sources based on different colors. The PubMLST ID and GenBank accession numbers of 10 ST220 *A. pittii* strains are shown as follows: AS012594 (ID: 6032, GenBank accession number: VLVU00000000), SU1805 (ID: 5840, GenBank accession number: JACWEV000000000), YB45 (ID: 5339, GenBank accession number: CP029610), AP984 (ID: 5927, GenBank accession number: JAEHOG000000000), AP864 (ID: 5924, GenBank accession number: JAEFCW000000000), NBRC110506 (ID: 5858, GenBank accession number: BBTY00000000), WCHAP100010 (ID: 5991, GenBank accession number: SGTJ00000000), WCHAP100007 (ID: 6001, GenBank accession number: SGTM00000000), WCHAP100022 (ID: 6006, GenBank accession number: SGSZ00000000), and NBRC110505 (ID: 5861, GenBank accession number: BBTX00000000).

We further queried the PubMLST database, and the information of 10 other ST220 *A. pittii* strains was obtained. Strains were collected from several different sources, including blood, sputum, abscesses, and the upper respiratory tract of patients and environmental sinks (**Figure 7**). Their hosts were isolated in various cities in the USA, Japan, China, and Thailand. Comparative genomics analysis showed that there is a very close relationship between the *A. pittii* strain YB45 and the *A. pittii* TCM strain used in this study. Moreover, the *A. pittii* AP864 and AP984 strains collected from Thailand were of the same branch. Furthermore, three strains isolated from Chengdu in China, namely WCHAP100010, WCHAP100007, and WCHAP100022, were closely related.

### Taxonomic relationship and pan-genome analysis

**Figure 8:**
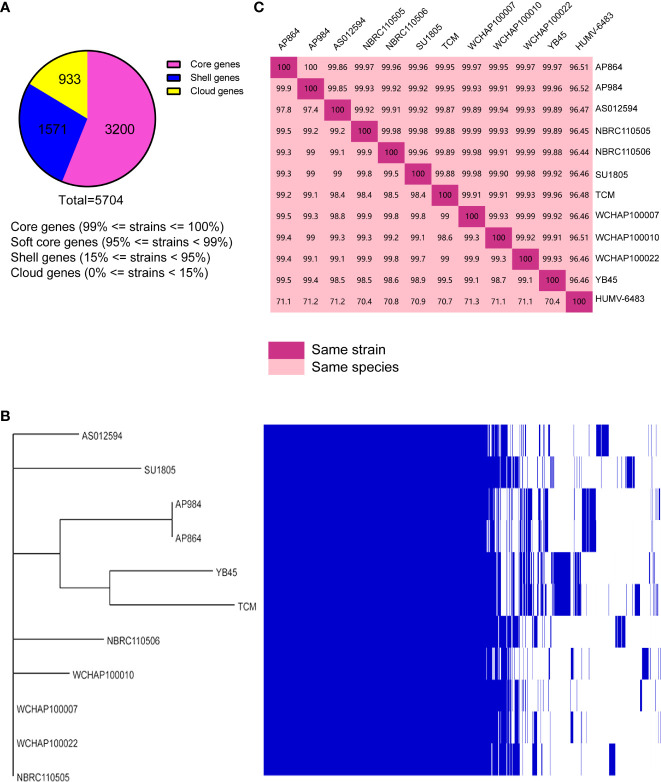
Pan-genome analysis of ST220 *A*. *pittii* strains using Roary. **(A)** Number of genes belonging to the core, the shell, or the cloud is shown as a pie chart. **(B)** Matrix of the presence/absence of genes generated with Roary and the phylogenetic tree of 11 ST220 *A*. *pittii* strains is also shown. The figure was visualized using Phandango. **(C)** Matrix distance values among *A*. *pittii* strains. The upper part shows average nucleotide identity (ANI) values; the lower part indicates DNA–DNA hybridization (DDH) values. A reference strain, namely *A*. *pittii* HUMV-6483, is also included to confirm the bacterial species. The threshold to belong to the same species is considered to be 95% for ANI values, whereas >70% is the threshold for DDH values. Diagonal 100% means the same strain.

Public genomes were reannotated using Prokka, and then a gene presence/absence analysis was performed on the basis of the annotated protein sequences. As shown in **Figure 8A**, 3,200 core genes, 0 soft core genes, 1,571 shell genes, and 933 cloud genes were defined. The presence/absence of all the genes were further visualized with the phylogenetic tree (**Figure 8B**). From that tree, the close relationship between *A. pittii* strain YB45 and *A. pittii* TCM strain was also observed.

To further compare the taxonomic relationship among all ST220 *A. pittii* strains and *A. pittii* HUMV-6483 reference strain, both ANI and DDH distances were calculated. All ST220 *A. pittii* strains presented high ANI and DDH values, ranging from 99.85% to 100% for ANI and from 97.4% to 99.9% for DDH (**Figure 8C**). Furthermore, the reference strain averaged 96.47% for ANI and 70.9% for DDH among the isolates used in this study (**Figure 8C**).

## Discussion

The presence of carbapenemase-producing *Acinetobacter* spp., including *A. baumanii*, *A. lwoffii*, and *A. pittii*, has become dominant in several countries, and they are increasingly being considered quite important nosocomial pathogens (Wisplinghoff et al., 2012; Mohd Rani et al., 2017; Mulani et al., 2019; Kiyasu et al., 2020). Although carbapenemases (especially for OXA-23, OXA-24/40, OXA-58) are widely disseminated among *Acinetobacter* species, few data are available for pathogenic *A. pittii* strains harboring carbapenemase except for sporadic reports (Sung et al., 2015).

Metallo-ß-lactamases (MBLs) (e.g., NDM-1) could neutralize the activity of ß-lactam antibiotics *via* hydrolyzing amide bonds. The spread of MBLs worldwide is the result of the lack of appropriate inhibitors and the transfer of the resistance genes that are located in the composite transposon structure. Hence, MBLs are known to support bacterial survival as powerful weapons against antibiotics (Behzadi et al., 2020). In previous studies, Yang et al. (2012) found MBL-producing *A. pittii* (NDM-1-positive) disseminated predominantly within the ICU in China. However, limited data and knowledge concerning NDM-1-positive *A. pittii-*causing BSI have been acquired to date in China (Yang et al., 2021). Authors’ study, one carbapenem-resistant *A. pittii* strain from BSI was isolated. To promote understanding regarding the genomic function of our *A. pittii* strain, the RAST software was utilized to classify the different CDS into subsystems according to their function (Aziz et al., 2008). Consistent with other published studies, the majority of the genes belong to “Metabolism,” followed by features with cellular components (amino acids, carbohydrates) (Chapartegui-Gonzalez et al., 2022).

Regarding the carbapenem-resistant *A. pittii* isolates, an outbreak of ST63 clone that carried a 45-kb *bla*_NDM-1_-bearing plasmid was reported in an ICU in China (Yang et al., 2012). In addition, Chopjitt et al. (2021) reported the ST63, ST396, and ST220 carbapenem-resistant *A. pittii* strains from the perspective of clinical characteristics and genome-based single nucleotide polymorphism (SNP). However, no studies completely clarify the chromosome and plasmid structures of *bla*_NDM-1_-positive carbapenem-resistant *A. pittii* strain. Few studies highlight the importance of mobile genetic elements (MGEs) in *A. pittii*. MGEs, including insertion sequences (ISs), integrons, and transposons, play a particularly important role in the resistance gene transfer between plasmid and chromosomes (Gorbunova et al., 2021). One study from Chapartegui-González et al. (2022) revealed that various ISs were found in all five *A. pittii* isolates. In particular, IS*Aba125* was found in a HUMV0315 *A. pittii* strain that was collected from Santander, Spain. In the current study, we, for the first time, found *bla*_NDM-1_ in the chromosome that mediated two IS*Aba125-*based Tn*125* composite transposons, highlighting the importance of IS*Aba125-*mediated transfer of resistance determinants. The genetic structure of Tn*125* composite transposon was highly related (99.99% identity) to a previously described *bla*_NDM-1_-*bla*_OXA-58_-harboring plasmid from an *A. baumannii* strain isolated from the rectal swab of a hospitalized patient in an ICU in Hangzhou, China (Liu et al., 2021a). Considering that these two strains had the same geographic location, there is a possibility that Tn*125* transfers between the chromosomes and plasmids of two species *via* one translocation event. In addition to resistance gene transfer mediated by composite transposons, XerC, and XerD, site-specific tyrosine recombinases (XerC/D-like sites) and a 28-bp recombination site *dif*, could play key roles in the resistance genes transfer of *bla*_OXA-40-like_, *bla*_OXA-499_, and *bla*_OXA-58_ (Cameranesi et al., 2018). *A. pittii* could be a crucial source of resistant genes and lead to the dissemination of resistant genes among species.

The degree of *A. pittii* virulence remains poorly understood. *ompA*, *pgaABCD*, and *bfmRS* are identified in our strain, which are able to promote adhesion and biofilm formation (Geisinger et al., 2018). This is a crucial pathogenic feature of many bacteria, facilitating colonization on the surface of biological materials, leading to further medical device-associated infections and promoting the evasion of the host immune system *in vivo* (Gordon and Wareham, 2010; Harding et al., 2018). More importantly, many virulence factors encoding Acinetobactin were found. Conde-Pérez et al. (2021) uncovered the essential role of Acinetobactin in the pathogenicity. Additionally, OCL and KL gene clusters, which also represent the virulence factor and are responsible for the biosynthesis of the outer core of lipooligosaccharide and capsules, are potentially useful epidemiological markers and may perform a key role in vaccine and biomarker development (Wyres et al., 2020). In our study, KL38 had a quite high identity. Whether all *bla*_NDM-1_-positive carbapenem-resistant *A. pittii* strains belong to this kind of KL type still needs further study. There are 12 prophage regions identified in the chromosome. A previous study from Krahn et al. (2016) demonstrated that transposon Tn*125*, which embedded the *bla*_NDM-1_ gene, could transfer *via* phage-mediated transduction within the species of *A. baumannii*. Thus, prophages may play a key role in the horizontal gene transfer (HGT) of carbapenems resistance genes, such as *bla*_NDM-1_ and *bla*_OXA-23_ (Krahn et al., 2016; Abouelfetouh et al., 2022). Another study from Loh et al. (2020) in China showed a wide variation in the number of prophages in *A. baumannii* genomes. Several phages carry carbapenems resistance genes, including *bla*_NDM-1_ and *bla*_OXA-23_, demonstrating the importance of lysogenic phages in the transfer of resistance genes. However, an important limitation of our study is that the activities of these prophages were not confirmed through induced experiments for prophages.

*Acinetobacter* spp. have a relatively small genome size compared to the other Gram-negative pathogens Therefore, there are fewer CDS (Chapartegui-Gonzalez et al., 2021; Chapartegui-Gonzalez et al., 2022). We further analyzed the genome features of all ST220 *A. pittii* strains based on the public database, in particular with the NDM-1 type carbapenemase. Zhang and Zhou (2018) reported the draft genome sequence of an NDM-1-, OXA-421-, and AmpC-producing ST220 *A. pittii* YB45 strain in Anhui Province, China. Based on the comparative genomics analysis, there is a very close relationship between *A. pittii* strain YB45 and *A. pittii* TCM strain. These strains may be spreading among different provinces in China, and they require early recognition and detection of carbapenemases. All AP864, AP984, and SU1805 isolates collected from Thailand and Japan carried NDM-1. Therefore, we speculated that carbapenem-resistant *A. pittii* strains, which were isolated in Asia, seemed to be more likely to harbor NDM-1. These genome data might facilitate further understanding of the genomic feature of NDM-1-positive *A. pittii* strains.

This study provides a comprehensive pan-genome analysis. Among the analyzed strains, all ST220 strains have high ANI and DDH values, suggesting a close genetic relationship. The numerous genes on the core genome compared with the pan-genome also highlight the similarity among these sequenced strains. In other studies, pan-genome and core-genome sizes vary and possess pan-genome values between 3,000 and 6,500, similar to our genome data (Chapartegui-Gonzalez et al., 2021).

It is worth noting that current treatment options for carbapenem-resistant Gram-negative infections are limited and need new antibiotics (Bassetti et al., 2021; Tamma et al., 2021). Novel therapeutic options against resistant microorganisms such as cefiderocol, GSK-3342830, eravacycline, WCK 5153 with sulbactam, apramycin, and bacteriophage therapy should be considered to overcome the problematic Gram-negative pathogens (Isler et al., 2019). Additionally, high-dose sulbactam combined with either levofloxacin, minocycline, or tigecycline may promote superior rates of clinical improvement and clinical cure, especially for multidrug-resistant or extensively drug-resistant *Acinetobacter* spp. (Liu et al., 2021b).

## Conclusion

This study is the first to report co-producing chromosomal NDM-1 and OXA-820 carbapenemases in *A. pittii* collected from a BSI patient in China. This discovery highlights the clinical importance of this species. The complete structures of the chromosome and plasmids were analyzed. Tn*125* composite transposon could transfer *via* a translocation event, and this species also may disseminate among different provinces in China. Therefore, surveillance is warranted, and early detection of carbapenemase genes is recommended to avoid a major spread in healthcare settings, especially in the ICU.

## Data availability statement

The complete sequences of the chromosome of *A. pittii* TCM strain and plasmids pTCM-1, pTCM-2, pTCM-3, and pTCM-4 have been deposited in GenBank under accession numbers CP095407-CP095411, respectively.

## Ethics statement

This study was approved by the local Ethics Committees of the Hospital with a waiver ofinformed consent due to this study mainly focused on bacterial genome and the retrospective nature of the study.

## Author contributions

CT and MX designed the experiments, analyzed the data, and wrote the paper. CT, MX, LF, and YZ performed the majority of the experiments. MX isolated the bacteria. XF and SW supervised this study and reviewed and edited the paper. All authors read and approved the final version of the manuscript.

## Funding

This work was supported by the Medical Health Science and Technology Project of Zhejiang Provincial Health Commission (2022RC278) and the Natural Science Foundation of Zhejiang Province (LGF20H300003, LGF20H280002, and LQ19H160002).

## Conflict of interest

The authors declare that the research was conducted in the absence of any commercial or financial relationships that could be construed as a potential conflict of interest.

## Publisher’s note

All claims expressed in this article are solely those of the authors and do not necessarily represent those of their affiliated organizations, or those of the publisher, the editors and the reviewers. Any product that may be evaluated in this article, or claim that may be made by its manufacturer, is not guaranteed or endorsed by the publisher.

## Supplementary material

Supplementary Figure 1 PCR confirmation of *bla*_NDM-1_ and *bla*_OXA-820_. Colony PCR was performed on an *A. pittii* TCM strain and cultured without any antibiotics using specific primers. Blank is the no-template control.
