# Supplementary figures and images for: Emergence of uncommon KL38-OCL6-ST220 carbapenem-resistant Acinetobacter pittii strain, co-producing chromosomal NDM-1 and OXA-820 carbapenemases
Source: Front Cell Infect Microbiol. 2022 Aug 12;12:943735. doi: 10.3389/fcimb.2022.943735 (PMC9411868; doi:10.3389/fcimb.2022.943735)

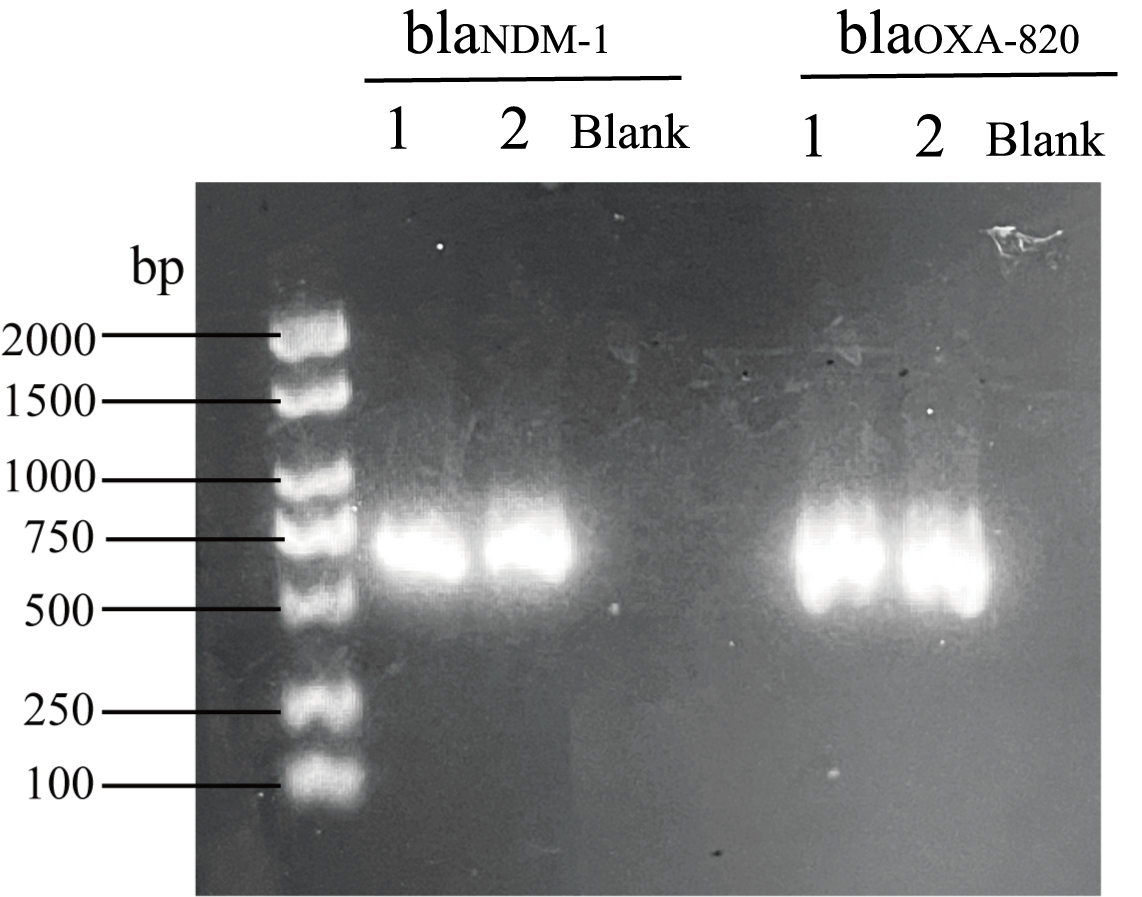

Supplement: Supplementary Figure 1 — PCR confirmation of bla NDM-1 and bla OXA-820. Colony PCR was performed on an A. pittii TCM strain and cultured without any antibiotics using specific primers. Blank is the no-template control. [file Image_1.tif]
