# Supplementary material for: Emergence of uncommon KL38-OCL6-ST220 carbapenem-resistant Acinetobacter pittii strain, co-producing chromosomal NDM-1 and OXA-820 carbapenemases
Source: Front Cell Infect Microbiol. 2022 Aug 12;12:943735. doi: 10.3389/fcimb.2022.943735 (PMC9411868; doi:10.3389/fcimb.2022.943735)
Supplement: Supplementary file 2 [file Table_1.docx]

**Table S1: Oligonucleotide primers used in this study**

| **Primer name** | **Sequence (5’-3’)** | **Length (bp)** | **Tm (℃)** | **References** |
| --- | --- | --- | --- | --- |
| *bla*_NDM-1_-F | TGGAATTGCCCAATATTATG | 690 | 55 | This study |
| *bla*_NDM-1_-R | CGGCGTAGTGCTCAGTGT |  |  |  |
| *bla*_OXA-820_*-*F | CGGTAGAACAGCATCAAATAC | 648 | 55 | This study |
| *bla*_OXA-820_-R | GAGGTTGAACGACCCAGC |  |  |  |

F, forward (5’) primer. R, reverse (3’) primer.
